# Comparison of surface roughness of root cementum and orthodontically induced root resorption craters from high- and low-fluoridation areas: a 3D confocal microscopy study

**DOI:** 10.1186/s40510-022-00415-6

**Published:** 2022-06-27

**Authors:** Chelsea Mann, Lam L. Cheng, Sarbin Ranjitkar, Selma T. Elekdag-Turk, Tamer Turk, M. Ali Darendeliler

**Affiliations:** 1 Department of Orthodontics and Paediatric Dentistry, Faculty of Medicine and Health, University of Sydney, Sydney Dental Hospital SLHD, Level 2, 2 Chalmers Street, Surry Hills, Sydney, NSW 2010 Australia; 2 Adelaide Dental School, University of Adelaide, Adelaide, Australia; 3 Department of Orthodontics, Faculty of Dentistry, Ondokuz Mayis University, Samsun, Turkey

**Keywords:** Fluoride, Root resorption, Cementum, Orthodontic force, Confocal microscopy

## Abstract

**Background:**

Fluoride has a major role in strengthening the structure of enamel against acids. Despite differences between caries and root resorption processes, both events inherently involve acidic dissolution of dental tissues. The aim of the present study was to investigate the effects of water fluoridation levels on the surface roughness of root cementum and resorption craters. The findings provided more insight into the influence of fluoride on the surfaces of intact cementum surface and resorption craters.

**Methods:**

Twenty-eight orthodontic patients were recruited from two cities in Turkey, with high (≥ 2 ppm) and low (≤ 0.05 ppm) water fluoridation. These patients needed bilateral maxillary first premolar extraction as part of their orthodontic treatment and were allocated into two study groups (*n* = 14 in each group) based on water fluoridation exposure level: the high-fluoride group (HF) and low-fluoride group (LF). 150 g of buccal tipping forces was applied to all maxillary first premolar teeth for 12 weeks with a beta-titanium spring which was reactivated every 4 weeks. All maxillary premolars were removed at the end of the experiment for surface roughness assessment using three-dimensional confocal microscopy and the associated software. The buccal root surface and the largest buccal resorption crater were investigated.

**Results:**

Resorption craters were significantly rougher in LF group compared to HF group (*p* = 0.002). Craters were rougher than the intact root surfaces (*p* = 0.000). Cervical and apical regions were significantly rougher than the middle region (*p* = 0.000 and *p* = 0.024, respectively).

**Conclusions:**

Higher water fluoridation level of ≥ 2 ppm resulted in significantly smoother root resorption craters than low water fluoridation level of ≤ 0.05 ppm when the teeth were subjected to 150 g of buccal tipping force. Fluoride seems to have a protective role at the interface of root resorption, and further mineral or histological studies may shed light on the exact protective process against root resorption.

## Background

Some degree of root resorption can be considered physiological as resorption craters have been reported in roots of unerupted third molar teeth [1]. Most (90.5%) non-orthodontic patients have some extent of root resorption [2]. The application of orthodontic forces can cause transient inflammatory surface resorption, otherwise known as orthodontic-induced inflammatory root resorption (OIIRR) [3]. Studies have reported an increase in root resorption in orthodontic patients from 15 to 73%, with moderate and severe root resorption increasing from 2 to 24.5% [4].

A decrease in susceptibility to OIIRR where cementum is more mineralised has been suggested [5, 6]. Fluoride has a major role in the prevention of dental caries by inducing the conversion of hydroxyapatite into fluorapatite and strengthening the structure of enamel against acids [7]. Despite differences between caries and root resorption processes, both events inherently involve acidic dissolution of dental tissues. In dental caries, the acid is produced externally by biofilm, whilst osteoclasts degrade bone and cementum by sealing a particular area with the ruffled border, delivering acid secretions as well as proteinases [8]. The acidic environment demineralises the inorganic components whilst providing an acidic environment for the optimal activity of the proteinases to degrade the organic material. The potential role of incorporating fluoride into cementum and its possibility of reducing root resorption are of interest. A few studies have investigated the effect of fluoride on OIIRR in rats [9–11] and humans [12, 13]. These studies demonstrated an inverse relationship between systemic fluoride exposure and root resorption crater size. One rat study also reported a decrease in the surface roughness of resorption craters and a reduction in the amount of tooth movement [14]. There are only two human studies that compared OIIRR in individuals from high and low water fluoridation areas after 28 days of light or heavy (25 g or 225 g) buccal orthodontic force [15, 16]. These studies reported significantly reduced volume of OIIRR with higher fluoride concentration (≥ 2 ppm) but only in the high force (225 g) group [15]. However, this effect on OIIRR was not found after 12 weeks of passive retention with multi-stranded stainless steel wire [16]. Studies relating fluoride to OIIRR is scarce, especially the lack of human studies reported in the literature.

The aim of the present study was to investigate the effects of water fluoridation levels on the surface roughness of root cementum and resorption craters in a clinically relevant human orthodontic model. The findings provided more information regarding the influence of fluoride on the interface at which root resorption occurs, the cementum surface and the resorption craters themselves. The null hypothesis was that there was no difference in surface roughness on root cementum and root resorption craters between subjects exposed to higher water fluoridation of ≥ 2 ppm and lower water fluoridation of ≤ 0.05 ppm.

## Materials and methods

The sample and selection criteria have been described in our earlier study [17]. Briefly, the sample comprised of 5 males and 23 females (average age 15 years, range 11–21 years) who required orthodontic treatment involving bilateral maxillary first premolar extraction. The subjects were recruited from two Turkish cities, including Isparta (high-fluoridation level of ≥ 2 ppm) and Samsun (low-fluoridation level of ≤ 0.05 ppm). The selection criteria were no significant medical history or craniofacial anomaly, no previous dental or orthodontic treatment on the maxillary first premolars (with complete apexification), no past or present sign or symptom of periodontal disease or bruxism and residence in the cities from birth (without migration). Ethics approval (2008/166) was granted by Ondokuz Mayis University in Turkey for this study, and specimens were analysed at The University of Sydney, Australia.

**Fig. 1:**
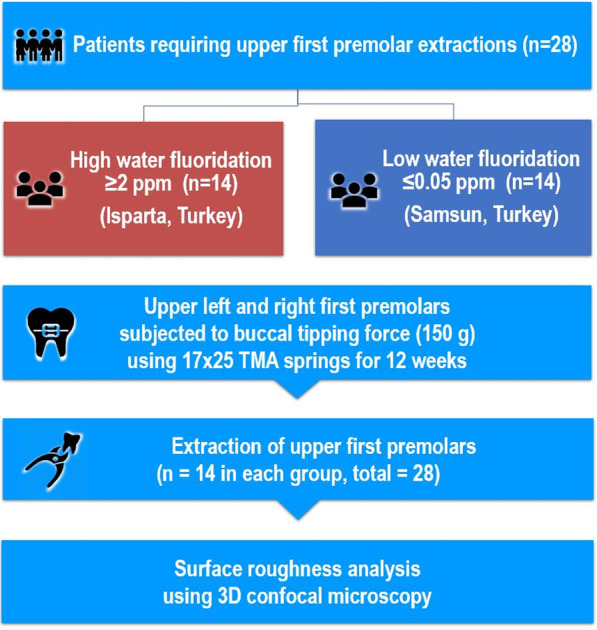
Flow chart of study design

**Fig. 2:**
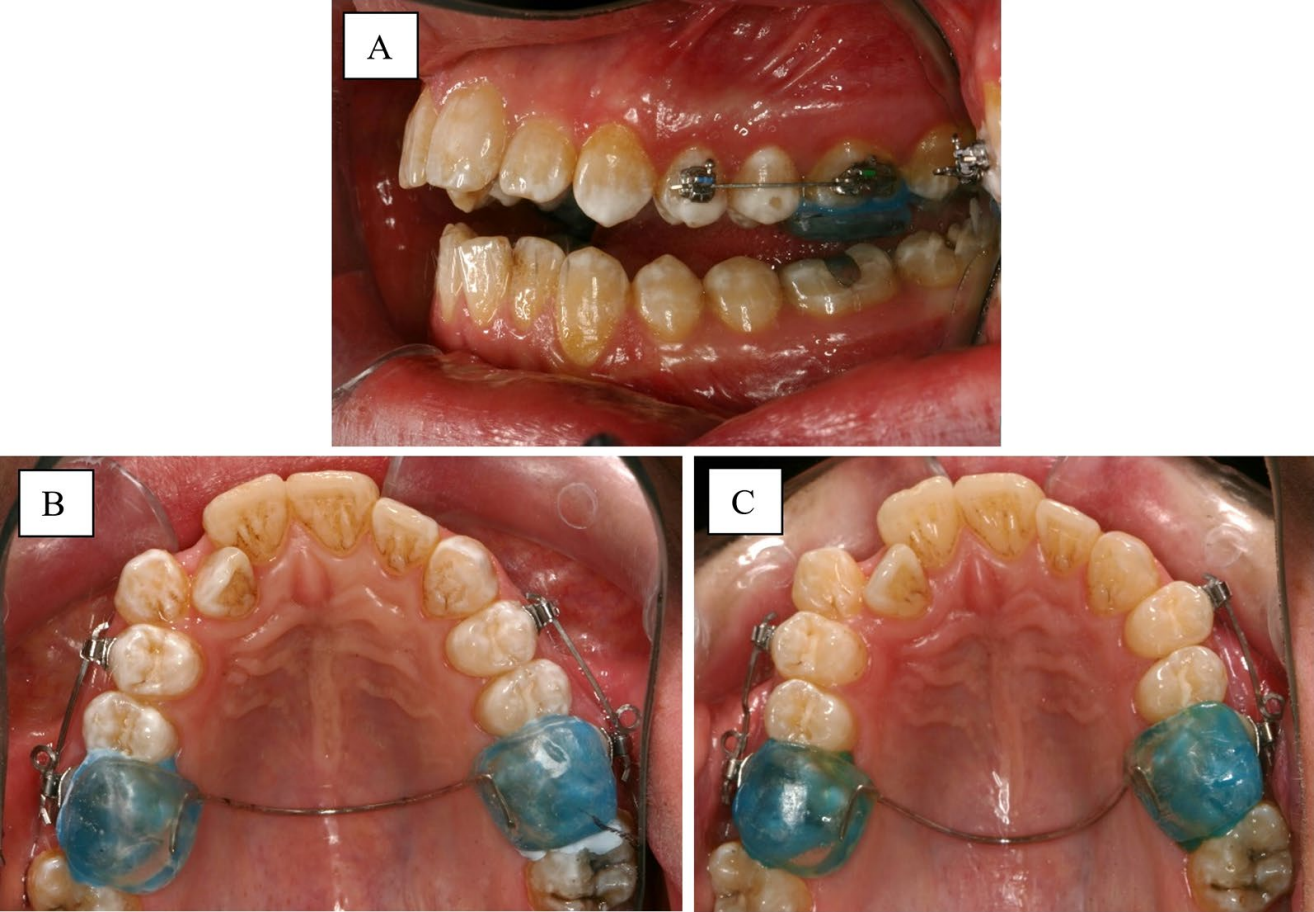
Intra-oral photographs of orthodontic appliances. **A** Buccal view on the day of appliance insertion, **B** occlusal view on the day of appliance insertion, **C** occlusal view after 12 weeks of buccal tipping force

The subjects were divided into high-fluoride group (HF) and low-fluoride group (LF) (Fig. 1). The buccal surfaces of maxillary first premolar and first molars were bonded with Speed brackets 0.022″ slot (Strite Industries, Cambridge, Ontario Canada). The ligation method was standardised by the nature of self-ligating brackets. To minimise occlusal forces to the maxillary first premolars, a transpalatal arch with posterior acrylic bite ramps was placed (Transbond Plus Light Cure Band Adhesive, 3 M Unitek, Monrovia, California, USA) (Fig. 2). A buccal tipping force of 150 g was applied for 12 weeks by using 0.017″ × 0.025″ beta-titanium-molybdenum alloy (TMA) cantilever springs (Beta III Titanium, 3 M Unitek, Monrovia, California, USA) from the maxillary first molars to the maxillary first premolars (Fig. 2). The springs were then reactivated every 4 weeks, followed by removal of the appliance and extraction of maxillary first premolars (using the extraction forceps on tooth crowns only) at 12 weeks. The teeth were stored in deionised water (Milli-Q) at ambient room temperature. Prior to analysis, the root surfaces were cleaned with a sterile gauze soaked in deionised water (Milli-Q) and periodontal fibres were removed with tweezers.

**Fig. 3:**
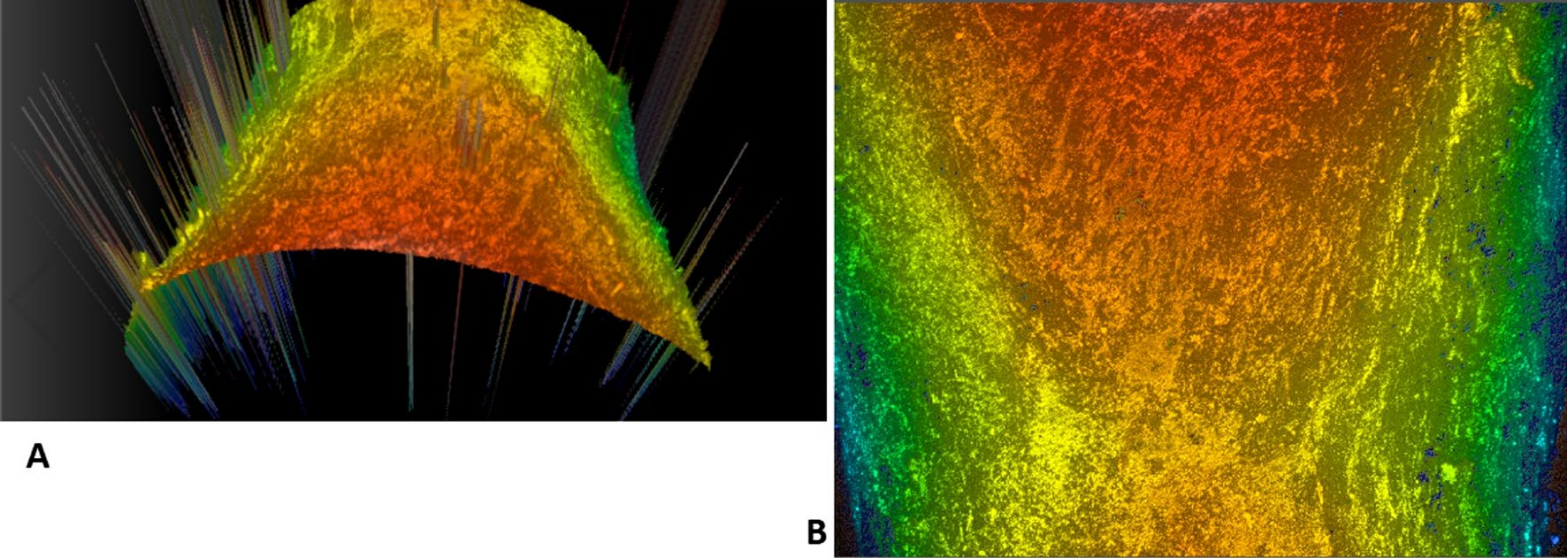
Example of raw data captured from three-dimensional confocal microscopy. **A** Three-dimensional view, **B** top-down view

**Fig. 4:**
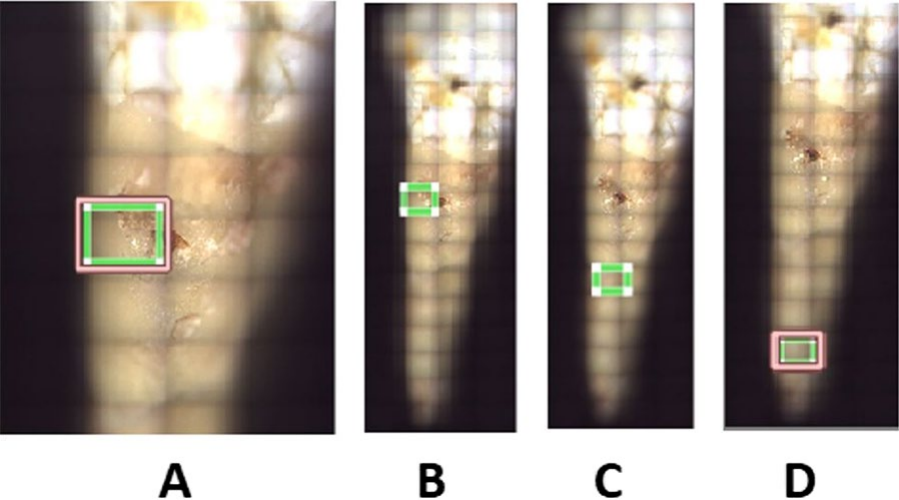
**A** Example area scanned with confocal microscopy, **B**–**D** example of scans taken at the buccal cervical, buccal middle and buccal apical regions of tooth root, respectively

Surface roughness was determined using a high definition three-dimensional confocal microscopy (Leica DCM8, Leica Microsystems, Germany) (Fig. 3). All roots were scanned at the largest buccal cervical root resorption crater and on the intact root surface cementum at the buccal cervical, buccal middle and buccal apical regions (Fig. 4). The largest buccal cervical root resorption crater was utilised as this was the most consistently present and identifiable crater. An exception was made of one sample in the LF group as there was no identifiable crater on the buccal cervical region and substitution was made with the largest palatal cervical crater. An optical lens of 10× magnification was used with no digital magnification to collect data from an area of 1.75 mm by 1.32 mm. The optical resolution was 0.46 microns. Specific software was used to extract surface roughness (Sa) values from the raw data (Mountains Map 7, Digital Surf, France). Curvature and spikes in the raw data determined to be more than 85 degrees to the flattened surface were removed prior to roughness calculations. Surface roughness values for craters were determined by two areas of 35 µm × 35 µm. The same two arbitrarily selected areas of 35 µm × 35 µm were used to calculate average surface roughness values. Exceptions were made in cases where resorption craters, cracks or obvious debris were present on the surface, whereby the nearest unaffected area was selected.

### Statistical analysis

Statistical Package for Social Sciences software (IBM SPSS Statistics for Windows, version 25.0, IBM, Armonk, NY) was used for statistical analysis. As the data and the residuals of the models were not normally distributed, log transformation was performed to obtain normality. One-way ANOVA was used to investigate the effect of fluoride, location and their interaction on surface roughness. Significance was set at the *p* < 0.05 level. Effect sizes using Cohen’s *f* were also calculated using the formula: $f = \surd \left( {\eta^{2} /\left( {1 - \eta^{2} } \right)} \right)$. An effect size (*f*) of 0.10 is mild, 0.25 is moderate, and 0.40 is high.

## Results

**Fig. 5:**
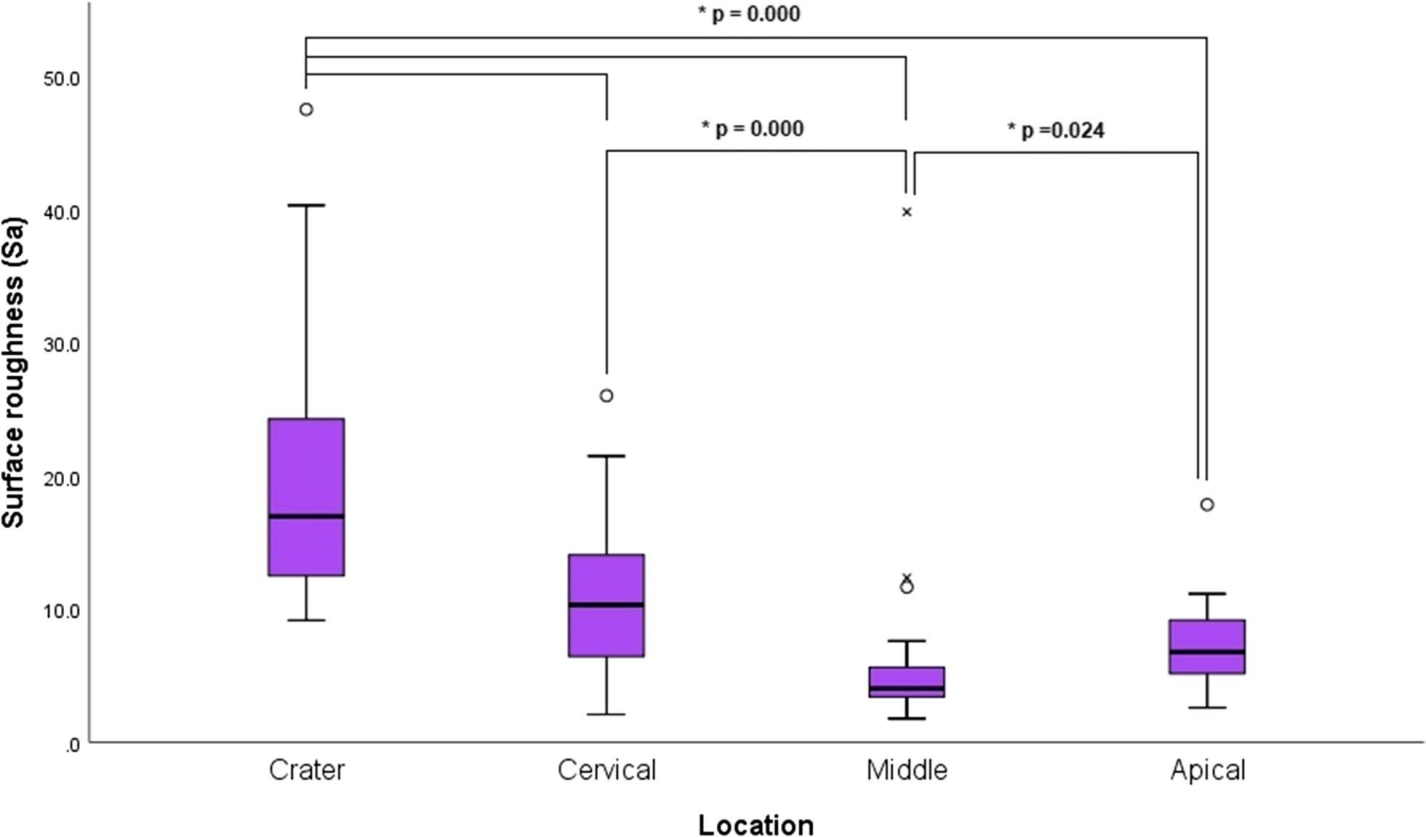
Box plot for surface roughness at different locations

Statistically significant differences were found between the locations and the interaction between fluoride and location. The root resorption craters were significantly rougher than the intact root surfaces (*p* = 0.000) (Fig. 5). The cervical and apical regions of the intact root surfaces were significantly rougher than the middle region (*p* = 0.000 and *p* = 0.024, respectively) (Fig. 5). The effect size was high (Table 2).

**Fig. 6:**
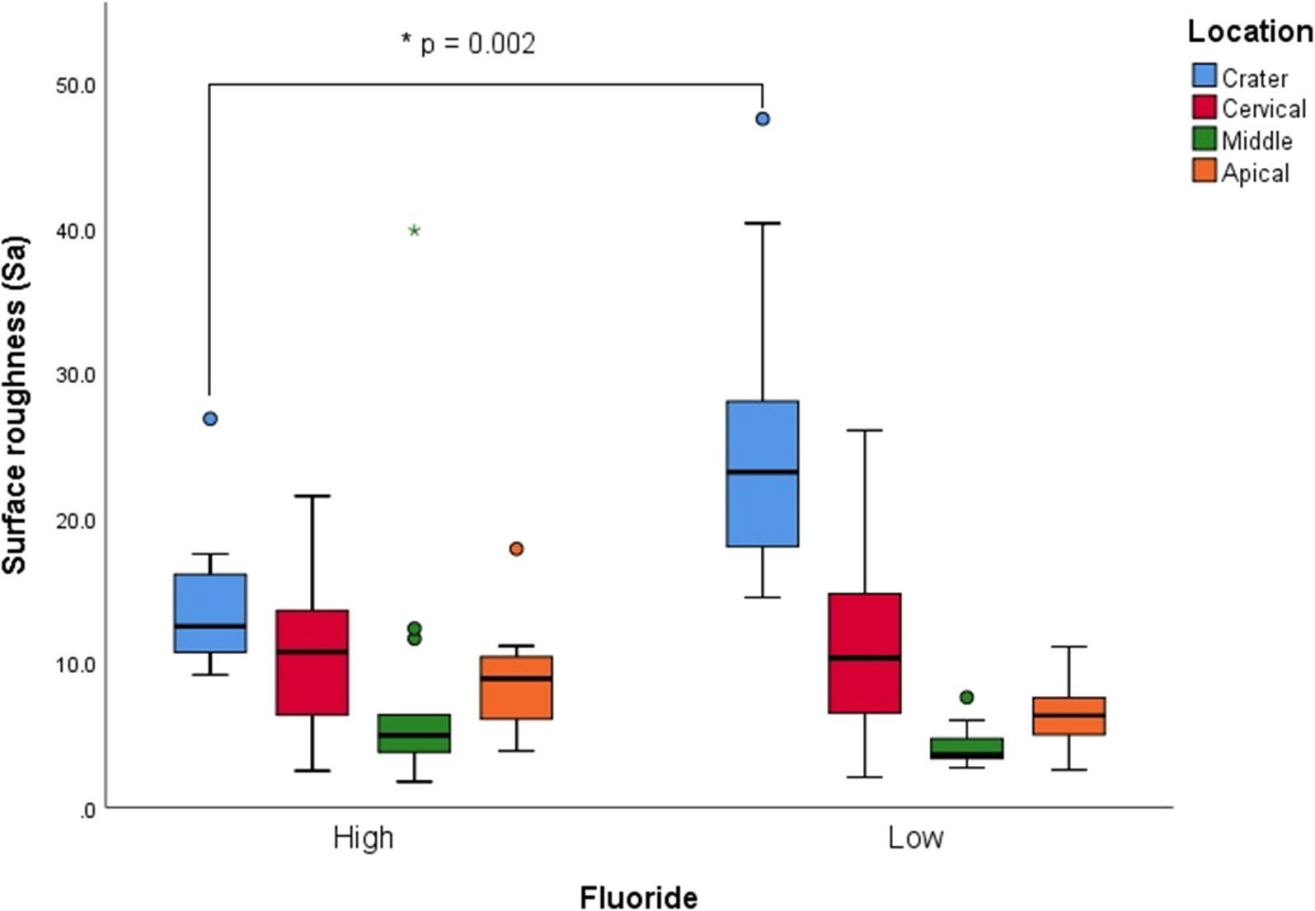
Box plot of surface roughness for high- and low-fluoride groups

At the resorption crater, the LF group was significantly rougher than the HF group (*p* = 0.002) (Table 1, Fig. 6). The effect size was high (Table 2).

**Table 1:** Surface roughness values

Location	Surface roughness (Sa)				Mean difference (95% CI)	*P* value*
	High fluoride		Low fluoride			
	Mean	SD	Mean	SD		
Crater	13.90	4.55	24.74	9.32	10.84 (5.14, 16.54)	0.002
Cervical	10.58	5.37	11.01	6.37	0.42 (−4.15, 5.00)	0.268
Middle	7.838	9.72	4.17	1.35	−3.67 (−9.06, 1.71)	0.967
Apical	8.69	3.57	6.32	2.32	−2.37 (−4.70, −0.03)	0.108
Total	10.25	6.52	11.56	9.85	1.31 (−0.955, 3.568)	0.255

*P values from one-way ANOVA with log transformation of Sa values (logSa)

**Table 2:** Effect size for fluoride, location and their interaction

	Cohen’s *f*	Interpretation
Location	0.89	High
Fluoride	0.11	Mild
Location and fluoride	0.49	High

## Discussion

The protective effect of fluoride on dental caries has been well established. Water fluoridation has been implemented in most major cities within the optimal range of 1 ppm for the beneficial effects of caries prevention without the consequence of significant fluorosis [17]. The electronegativity of fluoride readily converts hydroxyapatite to fluorapatite when fluoride is present in mineralised tissues. The deposition of fluoride has been shown to be greater in teeth than bone, and especially high in cementum [18].

In addition, the presence of fluoride favours bone formation by having an anabolic effect on osteoblasts [19] as well as significantly reducing the number of osteoclasts at the pressure regions of the periodontal ligament [20].

Previous studies have shown that fluoride could reduce the size of root resorption craters [14, 15, 21]. The volumetric root resorption of the same sample of the current study has also been assessed [22]. Significantly less root resorption was found on the palatal, palatal apical and pressure zones of the root. The presence of more systemic fluoride in the HF group likely resulted in the formation of more fluorapatite which is more structurally stable than hydroxyapatite. As fluorapatite was more stable and acid resistant, this may be responsible for less resorption and smoother surfaces at resorption craters in the HF group. Similar results were found in a previous rat study [14] that displayed a decrease in the surface roughness of root resorption craters in the higher fluoridated group.

An assessment of the resorption process as described histologically by Brudvik and Rygh [23–25] provides an explanation for the findings in the current study. Creation of root resorption craters is initially at the periphery of necrotic periodontal ligament by mononucleated macrophage-like and fibroblast-like cells. After a few days, tartrate-resistant acid phosphatase (TRAP)-positive giant cells without a ruffled border arrive and continue to remove the central part of the necrotic tissue. These cells remove precementum underneath and nearby the necrotic area to expose mineralised cementum. Whilst the removal of necrotic periodontal ligament appears to be via phagocytosis, the resorption of cementum appears to be extracellular only as phagocytosed cementum is not found inside these cells. Areas subjected to more forces such as the cervical and apical regions may have had some degree of surface resorption. The inspection of these intact areas had shown increased surface roughness. Thus, it is logical that trends in the data showed increased surface roughness in cervical and apical regions which have had increased force compared to middle region, and increased risk of some microscopic surface resorptions despite the assessed cementum surfaces being ‘intact’.

Part of the difference in roughness may also be related to the distribution of periodontal fibre insertion points. The cervical and apical regions of cementum typically have more embedded periodontal ligament supporting (Sharpey’s) fibres in order to support loading of the tooth in the longitudinal direction [26]. Cervical regions also have further gingival fibre insertions. Thus, the trend for increased roughness in these areas may be attributed to this.

When complete removal of the thickness of mineralised cementum has occurred, multi-nucleated odontoclast cells with a ruffled border are found adjacent to underlying dentine [25]. As we assessed the largest buccal crater in the buccal cervical region of each root, it is most likely that the resorption craters have entered dentine. Once resorption is into dentine, the process of resorption is like bone. The ruffled border of odontoclast cells seals off a particular area and produces mass acidic secretions and proteinases to resorb mineralised tissue and the organic matrix [8]. This process of resorption can be likened to dental erosion whereby differential rates of resorption of organic and inorganic components result in increased surface roughness [27]. Thus, it is logical that resorption craters were rougher than intact root surfaces.

This study contributed to the gap in the current literature regarding fluoride and orthodontic root resorption. The surface roughness which results from the resorption crater indicated that fluoride may have a protective role at the structural level. However, the exact mechanism is still unknown. It may be due to the incorporation of fluoride leading to stronger fluorapatite, or possibly enhanced recruitment of cementoblasts leading to thicker cementum and better repair of root resorption craters. Further investigation on the mineral contents and histological analysis may shed light on the process by which fluoride can protect teeth against OIIRR.

There may be a potential role for fluoride supplements during orthodontic treatment to increase systemic fluoride in low water fluoridation areas of < 0.05 ppm. However, the risk and benefit and the correct dosage of fluoride supplements require further investigation. Knowing systemic fluoride exposure influences root resorption and helps clinicians to identify a potential adjunctive preventive method for OIIRR.

## Conclusion

Exposure to higher water fluoridation seems to reduce the surface roughness orthodontic root resorption craters. Fluoride has a protective role at the interface of root resorption, but the exact mechanisms of action are still unknown. Further investigation on the mineral contents and histological analysis may shed light on the process by which fluoride can protect teeth against OIIRR. Furthermore, water fluoridation within the therapeutic level for dental caries with less risk of fluorosis needs to be tested.

## Data Availability

The datasets used and/or analysed during the current study are available from the corresponding author on reasonable request.

## Abbreviations

OIIRR: Orthodontic-induced inflammatory root resorption
HF: High-fluoride group
LF: Low-fluoride group
TMA: Beta-titanium-molybdenum alloy
Sa: Surface roughness
TRAP: Tartrate-resistant acid phosphatase
